# Esophageal Atresia with Tracheo-Esophageal Fistula Presenting Beyond 7 Days

**DOI:** 10.21699/jns.v6i3.577

**Published:** 2017-08-10

**Authors:** Nilesh Nagdeve, Mohini Sukhdeve, Tushar Thakre, Suresh Morey

**Affiliations:** 1 Department of Pediatric Surgery, Government Medical College, Nagpur.; 2 Department of Pediatrics, Government Medical College, Nagpur; 3 Department of Preventive and Social Medicine, Government Medical College Nagpur

**Keywords:** Esophageal atresia with trachea-esophageal fistula, Late presentations, Natural history

## Abstract

Aim: To describe our experience of neonates with esophageal atresia with tracheo-esophageal fistula (EA with TEF) who presented after a week.

Design: Retrospective study of the patients of EA with TEF who presented after a week.

Study Setting: Department of Pediatric Surgery, Government Medical College Nagpur.

Study Duration: Eight years.

Materials and Methods: Demographic information, hematological, biochemical and radiological data were obtained from the patients' medical records. The gap between two ends of the esophagus, nature of upper pouch and lower esophagus were noted intra-operatively. Outcome in terms of mortality and surgical complications were noted. In operated group, babies who survived were compared with non-survivors with respect to various preoperative variables.

Results: Of 52 patients, 27 babies expired during initial stabilisation period before surgery. The causes of mortality were severe pneumonitis and septicemia. One baby had associated cyanotic heart disease. Twenty-five patients with mean age of 8.28±1.21 days underwent surgery. Nearly two-third of them were male. All of them were born at full-term with mean birth weight of 2.47±0. 12 kg. More than 80% were previously hospitalised and nearly 70% babies were given feeds before present hospitalization. Mean Downe’s score for respiratory distress was 5.8±1.49. All patients were positive for septic profile. Associated congenital anomalies were present in ten patients. Intra-operatively, two ends of esophagus were either approximating or have short gap in 24 patients. All patients had well developed, thick and muscular upper oesophageal pouch. Lower esophagus at fistula was thin but dilated in 18 patients while thin and narrowed in 7 patients. However, esophageal anastomosis was possible with ease without any tension in all except one patient. There were 15 deaths in our study (13 due to pneumonitis and 2 during follow up due to aspiration). Three survivors required anti-reflux surgery. Comparison of preoperative variables of survivors and non-survivors showed a significant difference with respect to the variables like feedings, abdominal girth, immature band cells to neutrophil ratio and nature of pharyngeal or endotracheal aspirate.

Conclusions: Late presentations in EA with TEF are associated with high mortality but less anastomotic complications after surgery. Preoperative factors like feedings, abdominal distension, immature band cells to neutrophil ratio and bilious pharyngeal or endotracheal aspirate are associated with high mortality.

## INTRODUCTION

Esophageal atresia with tracheo-esophageal fistula (EA with TEF) is a well-known congenital anomaly with an incidence of 1 in 2,400–4,500 live births [1]. The abnormality was uniformly fatal throughout the world in first half of the twentieth century. Ever since Cameron Haight’s first report of successful surgical correction in 1941, the survival of neonates with EA with TEF has dramatically improved [2]. Now, the anomaly is regarded as an eminently correctable congenital lesion with survival rates more than 90% [3-5]. The improvement in survival rate is multifactorial and is largely attributed to the advances in neonatal intensive care, anaesthetic management, ventilatory support and surgical techniques over the past decades. Waterston proposed a prognostic classiﬁcation for EA with TEF in 1962 which included low birth weight, pneumonia and associated congenital anomalies as the risk factors [6]. The survival rates in Waterston’s study were 95%, 68% and 6% for group A, group B and group C, respectively. However, nowadays the survival can be achieved even in low birth weight babies [7], and the mortality is currently limited to those with coexistent severe life-threatening anomalies.

In the developed world these days, most of the cases of EA are diagnosed on antenatal sonography. However, issues related to public health services in third world countries like home deliveries, long distances between patients and health care centres or even the unawareness of the condition amongst primary treating medical personnel may contribute to the late presentation. There are isolated case reports of neonates with EA with TEF who presented late and survived after surgery [8,9]. Missing the diagnosis at birth and late presentation to tertiary care centre are the important factors that affect survival. The aim of this retrospective review is to describe our experience of neonates with EA with TEF who presented to us after a week.

## MATERIALS AND METHODS

This retrospective study was carried out in our department at Department of Pediatric Surgery, Government Medical College Nagpur and included patients of EA with TEF treated over an 8-year period, from January 2009 to December 2016. The study population consisted of the patients of EA with TEF who presented to us beyond 7 days of life.

Data were obtained from the patients' medical records and included information about age at presentation, sex, gestational age, birth weight, previous hospitalisation and feeds given and other important complaints like convulsions, apnoeic spells etc. Parameters like heart rate, respiratory rate, oxygen saturation, temperature, blood pressure, Downe’s score of respiratory distress [10], signs of shock, nature of pharyngeal or endotracheal aspirate (purulent, milk, hemorrhagic, bilious, or clear), abdominal girth and associated congenital malformations, were noted in all neonates. Babies were resuscitated and investigated. Hematological investigations included hematocrit, WBC count, platelet count, band cell to neutrophil ratio and prothrombin time. Biochemical investigations included serum creatinine, blood glucose, serum sodium, serum potassium, serum calcium, arterial blood gas analysis (ABG), serum bilirubin and C - reactive protein (quantitative assessment). Blood, CSF, pharyngeal/endotracheal aspirates were sent for culture and sensitivity in all patients. Radiological investigations like babygram (after putting catheter orally), ultrasonography of abdomen for associated other congenital and renal abnormalities and 2-D ECHO study were done in all neonates. All babies were grouped according to Waterston risk classification.

Management protocol included initial fluid resuscitation, broad-spectrum antibiotics (included anaerobic coverage), airway toileting (suction, nebulisation and chest physiotherapy). Downe’s score for respiratory distress was more than 6 and ABG decided the need of preoperative ventilation. Those who were in shock even after resuscitation were started on ionotropic support. Preoperative blood products transfusion were given in patients with hematological deficiencies.

The babies who survived, underwent primary repair after stabilisation. Surgery was done through the standard right postero-lateral thoracotomy and extra-pleural approach. Intra-operatively, the gap between two ends of the esophagus, the nature of upper pouch and lower esophagus were noted. The distance between the two ends of the esophagus was measured and was classified as approximating (<1cm,), the short gap (1-2cm), the intermediate gap (2-3cm) and the long gap (>3cm). The upper oesophageal pouch was grouped as either well developed, thick/muscular or atretic and as dilated or narrow. The esophagus at fistula was grouped as thick or thin and as wide (when a visible air leak was seen through it with esophageal distension during ventilation) or narrow (in absence of such findings). Postoperatively, standard management protocol was followed. Feeding was started through the trans-anastomotic feeding tube in the clinically stable patient, while in others partial parenteral nutrition was started. Antibiotics were started according to the culture reports or stepped up if needed; otherwise preoperative antibiotics were continued. Rest of the management was done according to the condition of the patient. In stable patients, contrast esophagogram was done on the 7th postoperative day. The patients were observed for any surgical complication in the postoperative period like minor or major anastomotic leak, recurrent trachea-esophageal fistula etc. The discharged patients were followed up for complications like apnoeic dying spell, recurrent pneumonia, failure to thrive, gastro-esophageal reflux (GER), recurrence of fistula or esophageal stricture. The contrast study was performed whenever parents gave the history like vomiting, severe episodic cough, apneic spells, failure to thrive etc. The follow-up was done either by personal visits of patients or telephonically. In the first year, all patients were called for follow-up every three monthly and in case of any problems. In the second year, the follow-up was done for 2-3 times and thereafter 1-2 times a year depending upon patients’ condition or in case of any problem. However, the patients living at far-away places were advised to consult local paediatrician/clinician every 4 monthly or in case of problems. At the time of visit, they were instructed to call us so that we can speak to their clinician.

For analysis, the patients were categorised into two groups depending upon whether they survived till surgery: Group-A patients who underwent surgical repair. These patients were further categorized into two groups: group 1 those who survived and group 2 those who expired in the postoperative period. Both groups were compared with respect to various parameters mentioned above. Group-B babies were those who succumbed during initial stabilization before surgery.

Continuous variables that were not normally distributed (e.g. age) were compared using the Manne Whitney U test while others were compared using Student’s t-tests. Categorical variables like complications etc. were compared using the Chi-squared test. For small numbers, Fisher’s exact test was used. All tests were two-sided and p values less than 0.05 were considered statistically significant. Statistical analysis was performed using Stata Software version 10.0.

## RESULTS

Of the 566 patients who were managed for EA with or without TEF, 52 (8.2%) presented after 7 days of life (Figure 1). 27 (51.93%) of these patients succumbed during initial stabilisation before surgery. These babies were born at full-term but five of them were of Waterston’s type C. All were positive for septic profile. Associated congenital anomalies were present in twelve patients (Table 1). More than one anomalies were present in 3 patients. The details of their characteristics are given in Table 2. All these babies required ventilatory support, while ionotropic support was needed in 17 babies. The causes of mortality were severe pneumonitis and septicemia. One baby had associated cyanotic heart disease.

**Figure:**
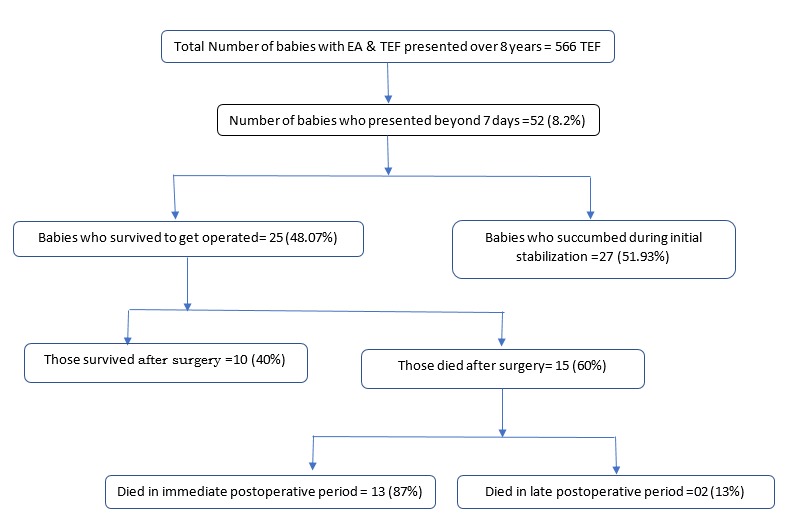
Figure 1: Flowchart showing the study population and outcome

Twenty-five (48.07%) patients who survived to get operated were analysed in details. The mean age of presentation was 8.28±1.21 days (range 7 to14 days) and nearly two-third of them were males. All of them were born at full-term (mean gestational age 36.08±0.97 weeks) with mean birth weight of 2.47±0.12 kg. One-fourth of them were born at home. More than 80% were previously hospitalised for complaints like not tolerating feeds, vomiting, respiratory distress, apnoeic spells, convulsions etc. Nearly 70% babies were given feeds before the present hospitalization. All babies were in respiratory distress at admission with mean Downe’s score of 5.8±1.49. All patients were positive for septic profile. Arterial blood gas analysis revealed mixed respiratory and metabolic acidotic picture in nearly two-thirds of the patients. Associated congenital anomalies were present in ten (40%) patients. Table 1 summarises the associated congenital anomalies. Demographic, hematological and biochemical parameters of our study population are shown in Table 2.

**Figure:**
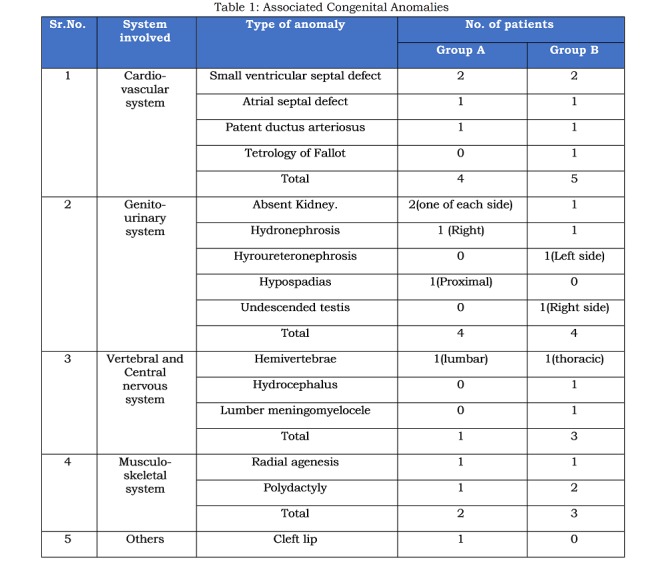
Table 1: Associated Congenital Anomalies

**Figure:**
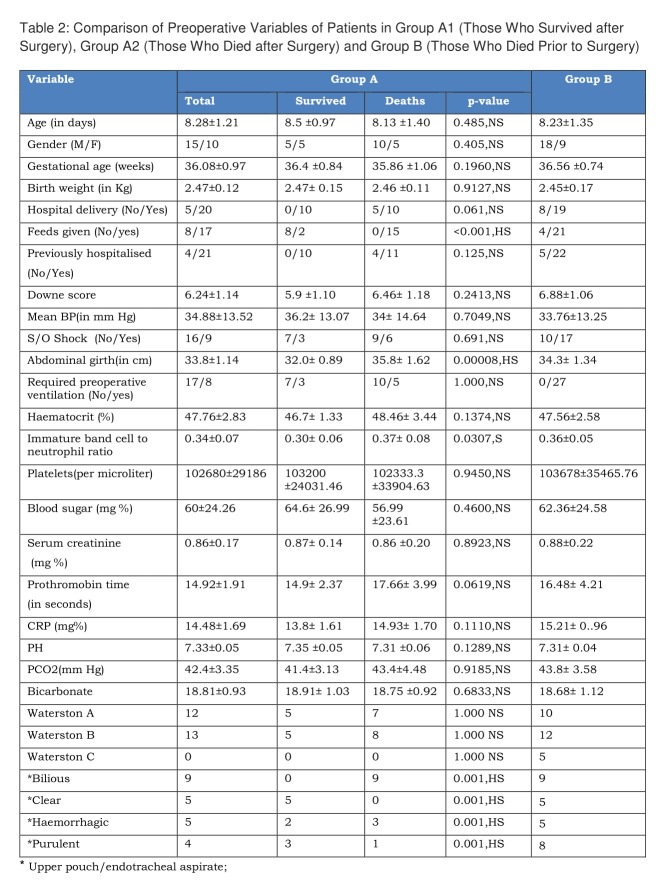
Table 2: Comparison of Preoperative Variables of Patients in Group A1 (Those Who Survived after Surgery), Group A2 (Those Who Died after Surgery) and Group B (Those Who Died Prior to Surgery)

Pharyngeal or endotracheal aspirates were bilious (44%), clear (20%), haemorrhagic (20%) or purulent (16%). However, positive cultures were present in 3 patients only (E.coli: n=2; Klebsiella: n=1). Blood cultures were positive in four patients (E. coli: n=2; Klebsiella and Staphylococcus 1each), while CSF culture were positive in two patients, both being E. coli. Pulmonary findings on chest radiograph consisted of patchy areas of consolidation (n=10), lobar consolidation (n=03) reticular shadows (n=08), and normal X-rays (n=4). The abnormalities were distributed bilaterally in 14 cases, while lobar consolidation involved right upper lobe.

The patients needed a period of 2.52±1.33days (range 0-6 days) for preoperative stabilization. The parameters of patients after initial stabilisation and just before surgery are shown in table 3. Eight patients required ventilatory support preoperatively. Nine patients who were in shock after initial resuscitation required ionotropic support, which was continued intra-operatively and postoperatively; the doses were adjusted according to patients’ parameters.

**Figure:**
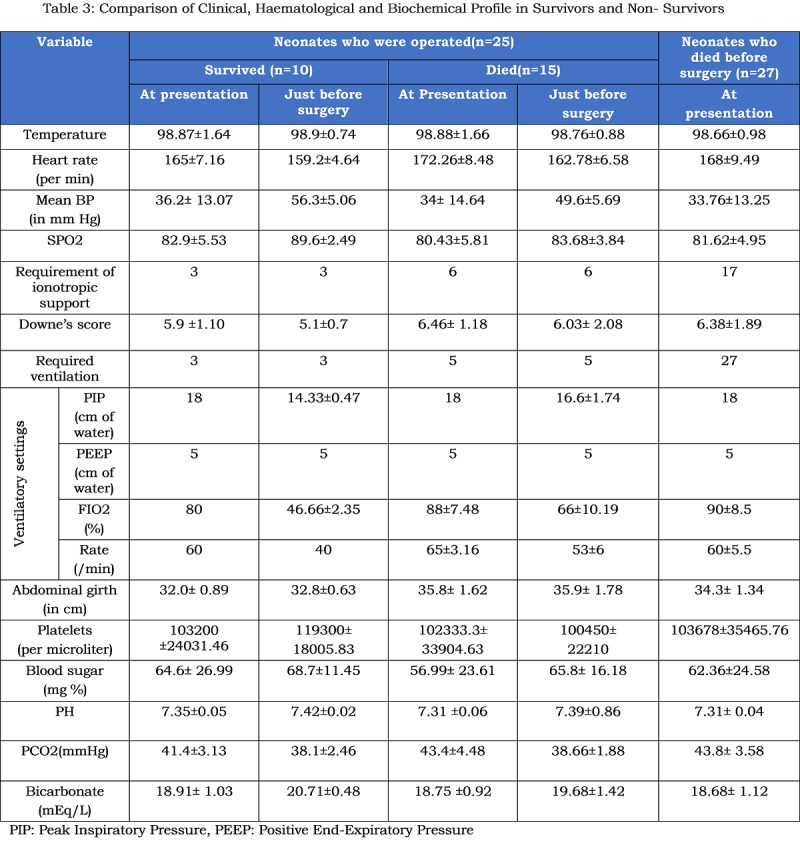
Table 3: Comparison of Clinical, Haematological and Biochemical Profile in Survivors and Non- Survivors

Intra-operatively, two ends of esophagus were approximating 12 patients. Twelve other patients had a short gap, while only one patient had a gap of 3 cm. All patients had well developed, thick and muscular upper oesophageal pouch. No one has proximal esophageal fistula. Lower esophagus at fistula was thin but dilated in 18 patients, while thin and narrow in 7 patients. None of the patient had well-developed, thick and muscular lower esophagus. However, esophageal anastomosis was possible with ease without any tension in all except one patient. Sixteen patients required postoperative ventilation.

There were 15 deaths in our study. Thirteen (87%) of the deaths occurred in early postoperative period, while the babies were still on assisted ventilation. The causes of death were severe pneumonitis and septicemia. Nine of these patients had survived for more than a week, but none had any demonstrable anastomotic leak as demonstrated. Two patients died after discharge in follow-up period due to aspiration on 68th and 87th day respectively.

All the patients were followed-up for a mean period of 2.84 years (range: 1.6-5.5 years). Of the ten survivors, five patients had upper gastro-intestinal contrast study done for symptoms like vomiting, episodic cough after a mean period of 4-months. Three of these required an additional surgery for severe. Thus, of the 12 patients who survived beyond 2 months, five (42%) had severe GER. Two of these died in follow-up period due to aspiration as mentioned above. Problems noted in five babies who were available for follow-up for more than 2 years, are shown in table 4. Complications like esophageal stricture or recurrent TEF were not seen on follow-up contrast study.

**Figure:**
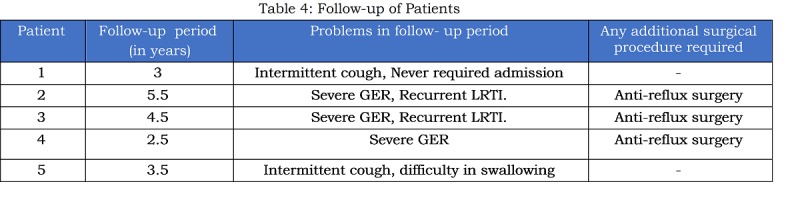
Table 4: Follow-up of Patients

Comparison of preoperative variables of patients who survived and those who succumbed is shown in table 2. Most of the variables do not exhibit significant difference in both groups. Statistically significant difference was found between two groups with respect to the variables like pre-operative feedings, abdominal girth, immature band cells to neutrophil ratio and nature of pharyngeal or endotracheal aspirate. Table 5 shows the correlation between nature of pharyngeal or endotracheal aspirate and survival in operated patients.

**Figure:**
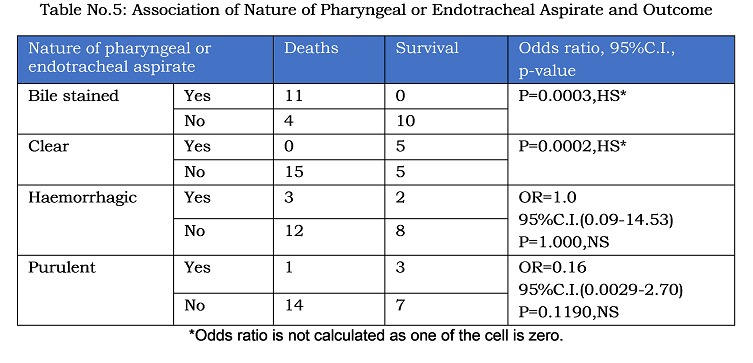
Table No.5: Association of Nature of Pharyngeal or Endotracheal Aspirate and Outcome

## DISCUSSION

The present study highlights an uncommon group of neonates with EA with TEF who presented to us beyond a week. Though such studies are a rarity nowadays, it has led to several significant observations, especially about the predictors of mortality in this group.

The reported incidence of EA with TEF in India is 18000 per year [11]. However, we feel that many of the cases go unreported. In our study, nearly 8% patients were diagnosed after a week. Though this appears to be a small number, this may be representing the tip of an iceberg. None of them had other major life threatening congenital anomaly and that they may be the reason that they survived that long. It also suggests that majority of the Waterston’s group B&C EA patients never reach the tertiary care hospitals. Such late presentations are due to poverty, ignorance, home deliveries and neonates from far-off places.

Another important observation made from this study is that 80% of the patients were either delivered at some hospital or attended by a medical practitioner who obviously missed the diagnosis of EA. To add to the insult, 70% of these babies were allowed feeds by qualified medical personnel. This points to the unmet need of creating awareness about the entity in medical fraternity.

Like Tandon et al., we also found male preponderance in these late presenters [12]. We agree with them that it is due to more preference to boys than girls in our society.

This group highlights few important anatomical aspects of EA with TEF. After initial stabilisation, the surgery was performed after a mean duration of nine days. In all except one, the distance between two ends of esophagus was less than two centimetres. Half of these babies had approximating oesophageal ends. Also, the upper oesophageal pouch was dilated, thick, and muscular. Probably the physiological growth and a stimulus of swallowing contributed to it. On the contrary, lower esophagus near fistula was thin in all babies but was dilated in three-fourth. None of the patients had well-developed lower esophagus. Lack of stimulus from swallowing or different organogenesis of lower esophagus as suggested previously, might be the causative factors [13]. However, none of our patients had anastomotic leak in post-operative period.

Studies have shown that apart from associated comorbidities and congenital anomalies, the complications and mortality is an outcome of gap length [14,15]. The long gap is associated with high incidence of anastomotic complication and other congenital malformations. A well-developed upper esophageal pouch and narrow gap between two ends of esophagus in our ‘delayed operated subset’, probably resulted in less anastomosis related complications.

Subgroup analysis revealed that preoperative factors like feedings, abdominal girth, immature band cells to neutrophil ratio and nature of pharyngeal or endotracheal aspirate resulted in statistically worse outcome. Most of these factors are avoidable. So, we tend to extrapolate that in a hospital delivered and carefully managed baby of EA with TEF, surgery can safely be deferred with good outcome. Abdominal distension and bilious aspirates in the absence of other gastrointestinal anomalies, is suggestive of wide tracheo-esophageal fistula.

The mortality in the Group B was 60%. The cause of death in these babies was severe pneumonitis and septicaemia which was similar to babies who succumbed during initial stabilisation. As reported by others also, pneumonitis has an established association with mortality [12]. Severe pneumonitis itself may misguide clinician in diagnosis, leading to delayed diagnosis. Prevention of respiratory complication should be the cornerstone of these patients and it may actually curtail mortality in EA with TEF.

Two of our patients were died after two months of surgery due to severe reflux and aspiration. Esophageal dysmotility and GER are common sequelae in patients who have undergone repair of EA with TEF. In our series, 30% of survivors required surgery for severe GER. Thus, GER is a major concern in this group. Chittmittrapap et a [16] showed that GER could significantly increase the esophageal stricture rate. Though none of our patients had either esophageal stricture or recurrent TEF, they continued to have episodes of cough and swallowing problems. We advocate early detection and surgical correction of GER to reduce postoperative morbidity and mortality.

Long-gap atresia is a problem as well as a challenge to the pediatric surgeons as it requires modifications from the conventional operation and has a high morbidity and mortality. Also, in today’s era EA with TEF is repaired thoracoscopically. Thus, in these scenarios, delaying a surgery by few days in properly selected patients may be advantageous. Tandon et al also shared a similar view as they observed that the age at the time of admission is not a bad prognostic factor [12]. Whether it is also applicable to high risk groups like Waterston’s group B and C or Spitz group II and III, is a matter of further study.

The main limitation of this study is that we have not compared this group with controls who presented early, as our aim was to focus on this rare group.

## CONCLUSION

To conclude, late presentations in EA with TEF are associated with high mortality rates, though the incidence of anastomotic complications was not high. Preoperative factors like feedings, abdominal distension, immature band cells to neutrophil ratio and bilious pharyngeal or endotracheal aspirate are associated with high mortality.

## Footnotes

**Source of Support:** None

**Conflict of Interest:** None

## References

[1] Myers NA. Oesophageal atresia: the epitome of modern surgery. Ann R Coll Surg Engl. 1974; 54:277–87. PMC2388409 4599652

[2] Haight C, Towsley HA. Congenital atresia of the esophagus with tracheoesophageal fistula: extrapleural ligation of fistula and endto-end anastomosis of esophageal segments. Surg Gynecol Obstet. 1943; 76:672–88.

[3] Spitz L. Esophageal atresia: past, present, and future. J Pediatr Surg. 1996; 31:19-25. 4. 10.1016/s0022-3468(96)90313-9 8632277

[4] Lopez PJ, Keys C, Pierro A, Drake DP, Kiely EM, Curry JI, et al. Oesophageal atresia: improved outcome in high-risk groups? J Pediatr Surg. 2006; 4:331-4. 10.1016/j.jpedsurg.2005.11.009 16481246

[5] Driver CP, Shankar KR, Jones MO, Lamont GA, Turnock RR, Lloyd DA, et al. Phenotypic presentation and outcome of esophageal atresia in the era of Spitz classiﬁcation. J Pediatr Surg. 2001;36:1419-21 10.1053/jpsu.2001.26389 11528619

[6] Waterston DJ, Carter RE, Aberdeen E. Oesophageal atresia: trachea-oesophageal ﬁstula. A study of survival in 218 infants. Lancet. 1962; 1:819-22. 10.1016/s0140-6736(62)91837-8 14005235

[7] Spitz L, Kiely E, Brereton RJ. Esophageal atresia: five year experience with 148 cases. J Pediatr Surg. 1987; 22:103–8. 10.1016/s0022-3468(87)80420-7 3820001

[8] Yadav K1, Nayar PM, Patel RV. Successful management of late presentation neonatal oesophageal atresia with distal tracheo-oesophageal fistula and significant pulmonary complications. Aust Paediatr J. 1987; 23:247-8. 10.1111/j.1440-1754.1987.tb00260.x 3426459

[9] Ansari A, Patel D, Joshi R, Chandana S, Bhattacharjee N, Sheth K. A 21 day old neonate with tracheo-esophageal fistula survived! - an all time record!! Gujarat Med J. 2009; 64: 89-90.

[10] Wood DW, Downes’ JJ, Locks HI. A clinical score for the diagnosis of respiratory failure. Amer J Dis Child. 1972; 123: 227-9 10.1001/archpedi.1972.02110090097011 5026202

[11] Upadhayaya P. Esophageal atresia in India. In: Willital GH, Nihoul-Fekete C, Myers NA, editors. Management of esophageal atresia: Diagnosis, therapy, complications and late results. Munich: Urban and Schwarzenberg; 1990. pp. 28–32.

[12] Tandon RK, Sharma S, Sinha SK, Rashid KA, Dube R, Kureel SN, et al. Esophageal atresia: Factors influencing survival - Experience at an Indian tertiary centre. J Indian Assoc Pediatr Surg. 2008; 1: 2–6. 10.4103/0971-9261.42564 PMC2810819 20177477

[13] Crisera CA, Connelly PR, Marmureanu AR, Colen KL, Rose MI, Li M, et al. Esophageal atresia with tracheoesophageal fistula: suggested mechanism in faulty organogenesis. J Pediatr Surg. 1999; 34:204–208. 10.1016/s0022-3468(99)90258-0 10022173

[14] Upadhyaya VD, Gangopadhyaya AN, Gupta DK, Sharma SP, Kumar V, Pandey A, et al. Prognosis of congenital tracheoesophageal fistula with esophageal atresia on the basis of gap length. Pediatr Surg Int. 2007; 23: 767-71. 10.1007/s00383-007-1964-0 17579871

[15] Brown AK, Tam PK. Measurement of gap length in esophageal atresia: A simple predictor of outcome. J Am Coll Surg. 1996; 182:41–5. 8542088

[16] Chittmittrapap S, Spitz L, Kiely EM, Brereton RJ. Anastomotic stricture following repair of esophageal atresia. J Pediatr Surg 1990; 25:508–11. 10.1016/0022-3468(90)90561-m 2352084

